# Supplementary material for: Molecular and Morphological Divergence of Australian Wild Rice
Source: Plants (Basel). 2020 Feb 10;9(2):224. doi: 10.3390/plants9020224 (PMC7076673; doi:10.3390/plants9020224)
Supplement: Supplementary file 1 [file plants-09-00224-s001.pdf]

Supplementary material 1

Table S1. Lists of core collections in NBRP (National Bioresource Project).

**Accession**	**Species**	**Origin (Collected site)**
W1297	*O. meridionalis*	Darwin, Australia
W1299	*O. meridionalis*	Darwin, Australia
W1300	*O. meridionalis*	Darwin, Australia
W1625	*O. meridionalis*	Darwin, Australia
W1627	*O. meridionalis*	Koolpinyah, Australia
W1631	*O. meridionalis*	Kununurra area, Australia
W1635	*O. meridionalis*	Darwin, Australia
W1638	*O. meridionalis*	Queensland, Australia
W2069	*O. meridionalis*	Kununurra area, Australia
W2071	*O. meridionalis*	Kununurra area, Australia
W2077	*O. meridionalis*	From Darwin to Normanton, Australia
W2079	*O. meridionalis*	From Darwin to Normanton, Australia
W2080	*O. meridionalis*	from Darwin to Normanton, Australia
W2081	*O. meridionalis*	Matarauka, Australia
W2100	*O. meridionalis*	Queensland, Australia
W2103	*O. meridionalis*	Queensland, Australia
W2105	*O. meridionalis*	Queensland, Australia
W2112	*O. meridionalis*	Queensland, Australia
W2116	*O. meridionalis*	Weipa, Queensland, Australia
Asian *O. rufipogon*		
W0106	*O. rufipogon*	Phulankara, near Cuttack, Orissa, India
W0107	*O. rufipogon*	Pahala, Orissa, India
W0108	*O. rufipogon*	Cuttack, Orissa, India
W0120	*O. rufipogon*	Cuttack, Orissa, India
W0137	*O. rufipogon*	Kadiam, Andhra, India
W0180	*O. rufipogon*	Ngao, Lamphang, Thailand
W0593	*O. rufipogon*	Binjai Rendah, Malaya
W0610	*O. rufipogon*	Rangoon, Myanmer
W0630	*O. rufipogon*	Magwe, Myanmer
W1294	*O. rufipogon*	Musuan, Mindanao, Philippines
W1551	*O. rufipogon*	Saraburi, Thailand
W1666	*O. rufipogon*	Siliguri, India
W1669	*O. rufipogon*	Orissa, India
W1681	*O. rufipogon*	Orissa, India
W1685	*O. rufipogon*	Orissa, India
W1690	*O. rufipogon*	Chiengrai, Thailand
W1715	*O. rufipogon*	China
W1807	*O. rufipogon*	Sri Lanka

W1852	*O. rufipogon*	Chiang Saen, Thailand
W1865	*O. rufipogon*	Saraburi, Thailand
W1866	*O. rufipogon*	Saraburi, Thailand
W1921	*O. rufipogon*	Saraburi, Thailand
W1939	*O. rufipogon*	Bangkoknoi, Thailand
W1945	*O. rufipogon*	No description
W1981	*O. rufipogon*	Palembang, Indonesia
W2003	*O. rufipogon*	From Pajani to Bombay, India
W2014	*O. rufipogon*	India
W2051	*O. rufipogon*	Hobiganji, Bangladesh
W2263	*O. rufipogon*	Cambodia
W2265	*O. rufipogon*	Laos, PDR
W2266	*O. rufipogon*	Laos, PDR
W2267	*O. rufipogon*	Laos, PDR
Australian *O. rufipogon*		
W2078	*O. rufipogon*	From Darwin to Normanton, Australia
W2109	*O. rufipogon*	Queensland, Australia
Papua New Guinean *O. rufipogon*		
W1230	*O. rufipogon*	New Guinea (Indonesia)
W1235	*O. rufipogon*	New Guinea (Indonesia)
W1236	*O. rufipogon*	Papua New Guinea
W1238	*O. rufipogon*	New Guinea (Indonesia)
W1239	*O. rufipogon*	New Guinea (Indonesia)

Table S2. Plastid genotypes of core collections and samples collected from natural populations.

		cp genotypes				
**Accession**	**Species / Type**	**INDEL1**	**INDEL11**	**INDEL13**	**INDEL18**	**INDEL19**
W1299	*O. meridionalis*	101	102	102	101	102
W1300	*O. meridionalis*	101	102	102	101	102
Jpn2	m-type (Sotowa et al. 2013)	101	102	102	101	102
Jpn3	m-type (Sotowa et al. 2013)	101	102	102	101	102
P26j	Not determined	101	102	102	101	102
P27L	Not determined	101	102	102	101	102
P126	Not determined	101	102	102	101	102
P7E	Not determined	101	102	102	101	102
P7L	Not determined	101	102	102	101	102
P8W	Not determined	101	102	102	101	102
P8R	Not determined	101	102	102	101	102
P10H	Not determined	101	102	102	101	102
P12	Not determined	101	102	102	101	102

P17	Not determined	101	102	102	101	102
P21	Not determined	101	102	102	101	102
P22	Not determined	101	102	102	101	102
P23	Not determined	101	102	102	101	102
P5N	Not determined	101	101	101	101	102
P5W	Not determined	101	101	101	101	102
P27S	Annual (Sotowa et al. 2013)	101	102	101	101	102
P6	Not determined	101	101	102	101	102
W1297	*O. meridionalis*	102	102	102	101	102
W1625	*O. meridionalis*	102	102	102	101	102
W1627	*O. meridionalis*	102	102	102	101	102
W1631	*O. meridionalis*	102	102	102	101	102
W1638	*O. meridionalis*	102	102	102	101	102
W2069	*O. meridionalis*	102	102	102	101	102
W2071	*O. meridionalis*	102	102	102	101	102
W2077		102	102	102	101	102
W2078	*O. rufipogon*	102	102	102	101	102
W2079	*O. meridionalis*	102	102	102	101	102
W2080	*O. meridionalis*	102	102	102	101	102
W2081	*O. meridionalis*	102	102	102	101	102
W2100	*O. meridionalis*	102	102	102	101	102
W2103	*O. meridionalis*	102	102	102	101	102
W2105	*O. meridionalis*	102	102	102	101	102
W1235	*O. rufipogon*	102	102	102	101	102
W1238	*O. rufipogon*	102	102	102	101	102
W1239	*O. rufipogon*	102	102	102	101	102
W2109	*O. rufipogon*	102	102	102	101	102
Jpn1	r-type (Sotowa et al. 2013)	102	101	101	101	102
P10L	Not determined	102	101	101	101	102
P26a	Perennial	102	101	101	101	102
P26b	Perennial	102	101	101	101	102
P26c	Perennial	102	101	101	101	102
P26d	Perennial	102	101	101	101	102
P26e	Perennial	102	101	101	101	102
P26f	Perennial	102	101	101	101	102
P26g	Perennial	102	101	101	101	102
P26h	Perennial	102	101	101	101	102
P26i	Perennial	102	101	101	101	102
P120	Not determined	102	101	101	101	102
W1635	*O. meridionalis*	102	101	101	101	102
P5O	Not determined	102	101	102	101	102

W2112	*O. meridionalis*	102	102	102	101	101
W2116	*O. meridionalis*	102	102	102	101	101
W1236	*O. rufipogon*	102	101	101	104	101
Nipponbare	*O. sativa*	102	101	101	102	101
W0120	*O. rufipogon*	102	101	101	102	101
W0106	*O. rufipogon*	102	101	101	103	101
W0137	*O. rufipogon*	102	101	101	103	101
W1230	*O. rufipogon*	102	101	101	103	101
